# Supplementary figures and images for: Blazing Signature Filter: a library for fast pairwise similarity comparisons
Source: BMC Bioinformatics. 2018 Jun 11;19:221. doi: 10.1186/s12859-018-2210-6 (PMC6047367; doi:10.1186/s12859-018-2210-6)

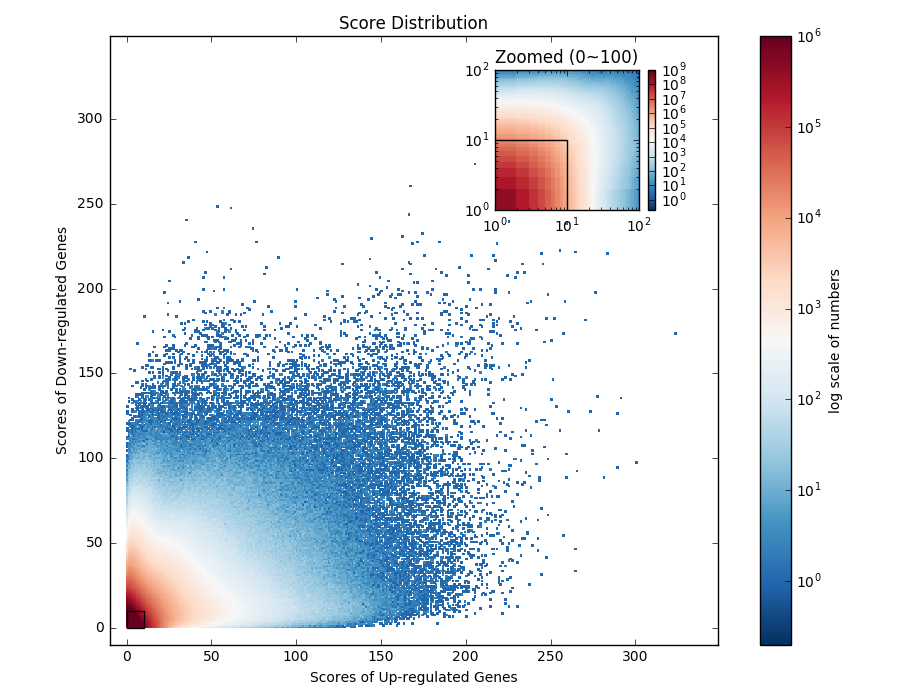

Supplement: Supplementary file 1 — Figure S1. Score distribution of the 6.89 billion pairwise comparisons in the LINCS L1000 dataset. The color of each point describes the number of pairs which have shared genes. X and Y axes indicate the number of shared up-regulated genes and down-regulated genes, respectively. For example, a point of (50, 50) has 147, which means 147 pairs of two signatures share 50 up-regulated genes and 50 down-regulated genes. The overwhelming majority of pairwise comparisons, ∼ 6.80 billions or 98.8%, are located in a small box of up-regulated genes < 10 and down-regulated genes < 10. These represent pairs of experiments, which do not share a discernable signature of regulated gene expression and are unproductive data mining events. (PNG112 kb) [file 12859_2018_2210_MOESM1_ESM.png]

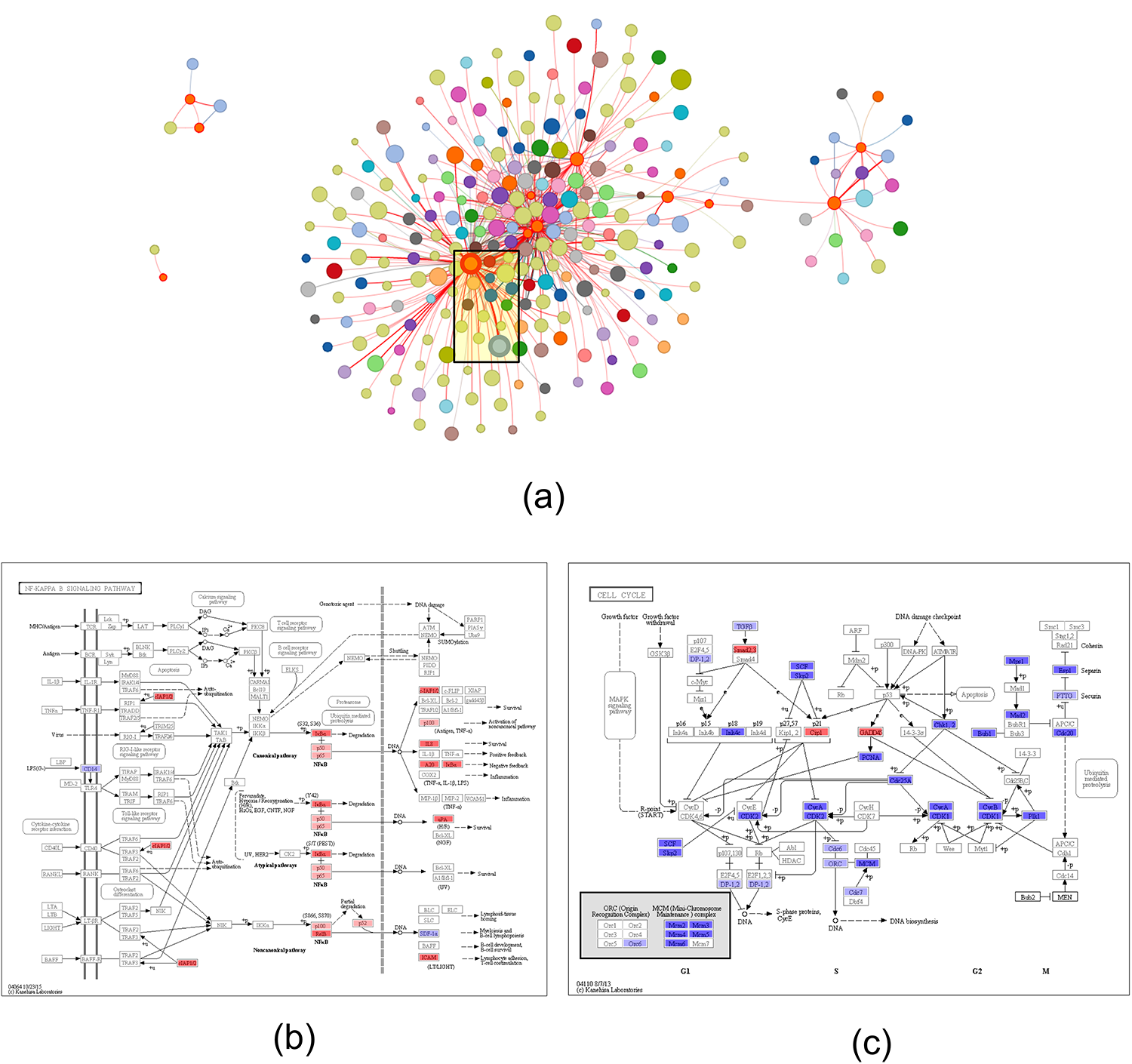

Supplement: Supplementary file 2 — Figure S2. A sub-network of LINCS L1000 experiments most similar to niclosamide. (a) We extracted the network for 88 datasets associated with non-human medications such as niclosamide (tapeworm infestations) and daminozide (plant growth regulator). It shows 257 experiments of 20 drugs highly connected to these 88 signatures. Refer to Materials and Methods for details. (b) Differentially expressed genes shared between niclosamide and IMD 0354, an IKKβ inhibitor. Most of all common genes are down-regulated and cell cycle looks slow down. (c) Shared differential genes shown for the NF- κB signaling pathway; most of the genes are up-regulated. (PNG 690 kb) [file 12859_2018_2210_MOESM2_ESM.png]
